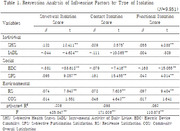# An Analysis of Social Isolation Types and Associated Influencing Factors in Older Adults: Focusing on Personal, Social, and Environmental Factors

**DOI:** 10.1002/alz70860_097709

**Published:** 2025-12-23

**Authors:** HYUN YANG, Suyeong Bae, Jiwon Shin, MuWon Lee, Hae Yean Park, ChaeYoung Lee

**Affiliations:** ^1^ Yonsei University, Wonju, Wonju, Korea, Republic of (South); ^2^ Yonsei University, Wonju, Korea, Republic of (South); ^3^ Graduate School, Yonsei University, Wonju, Wonju, Korea, Republic of (South); ^4^ College of Software and Digital Healthcare Convergence, Yonsei University, Wonju, Heungup‐meon, Korea, Republic of (South); ^5^ Yonsei University, Wonju‐si, Gangwondo, Korea, Republic of (South)

## Abstract

**Background:**

The purpose of this study is to identify the types of social isolation in the elderly in Korea and to analyze the effects of personal, social, and environmental factors on each type of isolation.

**Method:**

A secondary analysis was conducted on the data of 9,951 people using the 2023 Survey on Seniors. Social isolation of the elderly was classified into three types: structural isolation, contact isolation, and functional isolation, and multiple regression analysis was conducted to confirm the relationship between the types of social isolation and major factors.

**Result:**

Each type of structural isolation, contact isolation, and functional isolation showed significant correlation with variables such as subjective health status, electronic device utilization ability, and residence satisfaction. In particular, subjective health status, electronic device utilization ability, social participation satisfaction, and residence satisfaction were major influencing factors in all types of social isolation.

**Conclusion:**

This study proposes the necessity of developing customized interventions by analyzing in‐depth the categorization of social isolation and influencing factors of the elderly. In the future, through longitudinal studies and consideration of more diverse variables, it can be used as basic data for practical policy and program design to solve the problem of social isolation for the elderly.